# Predictors of prolonged hospitalization among children aged 2–59 months with severe community-acquired pneumonia in public hospitals of Benishangul-Gumuz Region, Ethiopia: a multicenter retrospective follow-up study

**DOI:** 10.3389/fped.2023.1189155

**Published:** 2023-07-06

**Authors:** Habtamu Dinku, Dessalegn Amare, Sileshi Mulatu, Melsew Dagne Abate

**Affiliations:** ^1^ Department of Pediatrics and Child Health Nursing, College of Medicine and Health Sciences, Bahir Dar University, Bahir Dar, Ethiopia; ^2^ Department of Adult Health Nursing, College of Health Sciences, Injibara University, Injibara, Ethiopia

**Keywords:** severe community-acquired pneumonia, time to recovery, children, prolonged hospitalization, Benishangul-Gumuz, Ethiopia

## Abstract

**Background:**

Pneumonia is a leading cause of morbidity and mortality among children aged under 5 years in Ethiopia. Prolonged hospitalization of severe community-acquired pneumonia is a significant problem in resource-limited countries. This study seeks to provide insights that can help improve the management and outcomes of severe community-acquired pneumonia, which is particularly important in the context of the Benishangul-Gumuz Region, Ethiopia, where access to quality healthcare services is limited, and childhood pneumonia is a significant health challenge.

**Objective:**

The aim of the study was to determine the predictors of prolonged hospitalization among children aged 2–59 months admitted with severe community-acquired pneumonia between 1 January 2016 and 30 December 2020 in the public hospitals in Benishangul-Gumuz Region, Ethiopia.

**Method:**

A retrospective follow-up study design was conducted among randomly selected samples of 526 children. Data were entered into EPI data version 4.6 and analyzed using STATA version 14.0. The Cox proportional hazard regression model was fitted to identify the independent predictors of prolonged hospitalization, and variables with a *p*-value <0.05 in the multivariable model were considered statistically significant.

**Results:**

The median hospital stay was 5 days (interquartile range 2–8 = 6). Approximately 149 (28.93%) children had prolonged hospitalization (>5 days) and the recovery rate from severe community-acquired pneumonia was 19.69 per 100 person-day observations. The significant predictors of prolonged hospitalization were as follows: having facility referral sources [0.79, 95% confidence interval (CI), 0.63–0.98]; a nutritional status of wasting (0.64, 95% CI, 0.44–0.94); anemia (0.65, 95% CI, 0.46–0.90); no identified hemoglobin level (0.53, 95% CI, 0.41–0.70); no identified blood film (0.65, 95% CI, 0.53–0.80); no chest x-ray investigation (0.81, 95% CI, 0.65–0.99); pulmonary effusion (0.31, 95% CI, 0.15–0.66); and late presenters to hospital (0.67, 95% CI, 0.53–0.84) at admission.

**Conclusions:**

The median length of stay in hospital was delayed compared to other studies. Wasting, late presenting to hospital, pulmonary effusion, anemia, absence of investigations of hemoglobin level, chest x-ray, and blood film at admission time were factors that significantly prolonged the hospitalization time. Hence, attention should be given to the prevention of malnutrition and anemia in children, increasing early health-seeking behavior in the community. Attention should be given to complications such as pleural effusion, and investigations, such as chest x-ray, hemoglobin levels, and blood films, should be performed when the child is admitted.

## Background

Pneumonia is an inflammation of the parenchymal tissue of the lung, such as the alveoli and bronchioles (1). The World Health Organization (WHO) defines community-acquired pneumonia in children as the presence of a cough or difficulty breathing associated with age-specific fast breathing or chest indrawing that begins outside the hospital or is diagnosed within 48 h after admission (2–4). Severe community-acquired pneumonia (SCAP) can be defined as community-acquired pneumonia plus at least one of the following signs: the inability to drink or breastfeed; feeling lethargic or unconscious; having convulsions; vomiting everything; severe respiratory distress; central cyanosis; or stridor in a calm child (4, 5). The WHO defined SCAP only on the basis of signs and symptoms obtained by visual inspection and on timing of the respiratory rate (6). The clinical features are not specific; no single symptom or sign is present for SCAP in children. As a result of the clinical diagnosis of SCAP, the time to recovery (TTR) may be long (7). In order to facilitate the time to recovery from SCAP among hospitalized children, different methods of diagnosis were needed, such as chest x-rays (8).

Globally, childhood SCAP is one of the leading causes of mortality and morbidity as well as hospitalization burden for children aged under 5 years (9). The incidence of child hospitalization related to SCAP was 11.9 million, with three million SCAP results in hospital admissions per year worldwide. Among these admissions, 99% of deaths occurred in low-income countries, including Ethiopia (10). Many programs or policies implemented to reduce child mortality and morbidity related to SCAP, such as the WHO’s Integrated Management of Neonatal and Childhood Illness (IMNCI) and Integrated Community Case Management (ICCM) guidelines, provide health workers with the methods of diagnosing SCAP. However, it remains the leading cause of morbidity, mortality, and hospitalization of children (11). Even though the solutions settled, many children are more likely to develop SCAP. This may be due to the lack of access to quality of care, lack of essential medicines in health facilities, and lack of international government-related opportunities to save a child with simple and low-cost interventions in primary facilities, thus resulting in higher severity of SCAP and prolonged hospitalization (12).

Ethiopia adopted the WHO guideline and extensively wrought to achieve sustainable development goals (SDGs) to end preventable child deaths (13). According to the Ethiopian Demographic and Health Survey (EDHS) 2016, childhood deaths related to pneumonia were 18%, and SCAP caused 38.6% of emergency hospital admissions, related bed occupancy, and healthcare cost burdens (14). The prolonged hospitalization of children related to SCAP has a psychological and emotional impact on both children and their families (15, 16). In addition, SCAP constitutes an enormous economic and social burden related to the prolonged hospitalization of children, especially in resource-limited countries (17).

In Ethiopia, studies were conducted regarding the time to recovery from SCAP (18–20). However, these studies were conducted in a single institutional study area and conducted over a shorter study period, whereas our study was conducted in six hospital study areas over a longer study period of 5 years, which increased the statistical power and generalizability of the study. In addition, our study area was remote and the majority of the population lives in a rural region with little access to healthcare and low levels of education, which will affect children recovering from SCAP in different aspects. Furthermore, the predictors of SCAP, such as radiographic findings, laboratory investigation, and referral sources, were included in our study and have not been addressed in previous studies. However, this might add value for the possible predictors of time to recovery from SCAP and indicate interventions to improve the program (19). Previous studies showed that predictors vary in contexts such as healthcare infrastructure, sociodemographic characteristics, and socioeconomic factors. Therefore, the aim of the present study was to determine the predictors of prolonged hospitalization among children aged 2–59 months admitted with SCAP in public hospitals in the Benishangul-Gumuz Region, Ethiopia.

## Materials and methods

### Study setting, design, and period

A retrospective follow-up study was conducted between 1 January 2016 and 30 December 2020 in the public hospitals in the Benishangul-Gumuz Region, Ethiopia. The region is located in the western part of the country and is 634 km from the capital, Addis Ababa. It has a population of approximately 1.2 million people and there are six public hospitals that provide medical care to children with severe community-acquired pneumonia. The data were collected between 17 March 2021 and 17 April 2021.

### Study participants

All children aged 2–59 months who were diagnosed with severe community-acquired pneumonia between 1 January 2016 and 30 December 2020 within 48 h of hospital admission were included in the study.

### Inclusion and exclusion criteria

Children aged 2–59 months and admitted for SCAP between 1 January 2016 and 30 December 2021 were included. However, children with incomplete records of date of admission and discharge, and a SCAP diagnosis received more than 48 h after admission to the hospital were excluded from the study.

### Sample size determination

The sample size was estimated with the double population proportion sample size determination method using EPI Info version 7 with a 95% confidence interval (CI), 80% power, and a 1:1 ratio of unexposed to exposed patients. The proportion of outcomes with exposure was 85% compared to 93% without exposure in a retrospective cohort study conducted at Debre Markos Referral Hospital (19). The final sample size of this study was 526.

### Sampling techniques and procedures

Simple random samplings were used to select participants for this study. There are six public hospitals that provide medical care to children with severe community-acquired pneumonia, with a total of 7,200 children aged 2–59 months diagnosed with SCAP between 1 January 2016 and 30 December 2020. A proportional allocation formula was applied to select study participants from each hospital based on their caseload. After the list of medical registration numbers was extracted from each hospital, the proportionally allocated sample sizes were selected using a computer-generated simple random sampling.

### Variables of the study

The dependent variable was the length of hospitalization (time to recovery) of SCAP. The independent variables included the following: sociodemographic factors (age, sex, residence, health insurance, and source of referral); nutritional status (stunting, wasting, and underweight); comorbidity [malnutrition, human immunodeficiency virus (HIV), anemia, tuberculosis]; clinical presentation (danger sign, any complication, and time elapsed before admission); investigation (laboratory and chest radiography); immunization status; and drug regimen.

### Operational definitions

Length of hospitalization (recovery time) is defined as the time from the date of admission to the date of discharge from the hospital.
Censored is defined as lost to follow-up, death, left against medical advice, absconded, and transferred out for any reason.
Survival status means the outcome of children, either cured, discharged, or censored.
Fully vaccinated is defined as being vaccinated as per Ethiopian EPI guidelines (21). Partially immunized is defined as not being vaccinated as per Ethiopian EPI guidelines with the absence of certain vaccinations (21).

### Data collection tools and procedures

A data extraction checklist was developed by the authors after reviewing different studies (18, 20, 22, 23). It includes sociodemographic, clinical, and investigation information, as well as drug regimens and immunization-related factors. Data were collected from patients’ medical charts using a pretested checklist. The medical charts of eligible children were retrieved based on their medical registration number identified from the log book in the pediatrics ward in each hospital. The data collection team consisted of chart finders from the chartroom, two BSc nurse data collectors, and one supervising master of public health (MPH) professional with previous data collection experience. The extracted data were coded to avoid duplication.

### Data management and data analysis procedures

The data were coded and entered into Epi Data Manager version 4.6 and exported to Stata version 14 for cleaning, checking, and analysis. Age, weight, and height were further exported to Emergency Nutrition Assessment (ENA)-SMART software to calculate weight for height (WFH) %, weight for age (WFA) %, and height for age (HFA) % Z-scores. Descriptive statistics were presented with frequency tables and graphs for the categorical variables and the continuous variables were reported with means [mean ± standard deviation (SD)] and medians [interquartile range (IQR)]. The Kaplan–Meier survival curve was used to estimate the median survival time and to identify the presence of a difference in recovery time/length of hospitalization among categorical variables. The Cox proportional hazard model assumption was checked using the Schoenfeld residuals test, Cox–Snell residual, and parallel assumption test. The association between the independent variables and the outcome variable was assessed by the Cox proportional hazard model. Variables with a *p*-value <0.2 in the bivariable model were a candidate for multivariable analysis. A 95% CI of the adjusted hazard ratio (AHR) was computed and variables with a *p*-value <0.05 in the multivariable model were considered statistically significant on the dependent variables.

### Data quality control

Before the data collection, a pretest was carried out in Pawe Hospital with 27 (5%) cases from the sample size from the charts that were registered 1 month before the study period; the charts were not included in the final sample size. Then, the necessary modifications were made to the checklist. The 1-day training was given to data collectors and supervisors before the actual data collection. The completeness of the collected data was checked on the site daily during data collection, with feedback from the supervisor and the investigators. In addition, the data were carefully entered and a double data entry was performed.

## Results

### Sociodemographic characteristics

Among the 526 charts reviewed, 515 charts met the enrollment criteria for the final analysis; 11 charts were excluded from the analysis due to incomplete data records. Out of 515 children, more than half (56.50%) were boys and 282 (54.76%) were from rural areas. The majority of participants (*n* = 350, 67.96%) were self-referred and the median age was 13 months (IQR = 8–24 months) (Table 1).

**Table 1:** Sociodemographic and baseline characteristics of children with severe community-acquired pneumonia admitted from 1 January 2016 to 30 December 2020 (*n* = 526).

Variable	Category	Frequency (%)
Age in months	2–12	258 (50.10)
	13–59	257 (49.90)
Sex	Male	291 (56.50)
	Female	224 (43.50)
Residence	Urban	233 (45.24)
	Rural	282 (54.76)
Family health insurance	Insured	253 (49.13)
	Not insured	262 (51.87)
Referral source	Self-referral	350 (67.96)
	Facility referral	165 (32.04)
Vaccination status	Fully vaccinated	350 (67.67)
	Partially vaccinated	23 (4.47)
	Up to date	129 (25.05)
	Non-vaccinated	13 (2.52)
Duration of illness before admission	Late presenter (>5 days)	151 (29.32)
	Early presenter (≤5 days)	364 (70.68)

### Clinical investigations and drug regimen characteristics

The most common nutritional problems were wasting (*n* = 54, 11.74%), stunting (*n* = 61, 11.60%), and underweight (*n* = 63, 12.23%). Anemia was a predominant co-morbid disease (*n* = 45, 8.74%) and chest indrawing was a dominant danger sign (*n* = 128, 24.85%). Approximately half (53.20%) and more than half (58.64%) of the patients had hemoglobin and blood film investigation results, respectively. Chest x-ray (*n* = 183, 35.53%), complete blood count (*n* = 226, 43.88%), hemoglobin level (*n* = 274, 53.20%), and blood film (*n* = 302, 58.64%) were carried out during the treatment period and the majority (64.85%) of patients were treated with crystalline penicillin (Table 2).

**Table 2:** Clinical investigation and drug regimen characteristics of children from January 2016 to December 2020 (*n* = 526).

Variable	Category	Frequency (%)	Variables	Category	Frequency (%)
WFH	Normal	461 (88.16)	Chest x-ray	Yes	183 (35.53)
	Wasting	54 (11.74)		No	332 (64.47)
HFA	Normal	454 (87.07)	Complete blood count	Yes	226 (43.88)
	Stunting	61 (11.60)		No	289 (56.12)
WFA	Normal	452 (87.77)	Hemoglobin	Yes	274 (53.20)
	Underweight	63 (12.23)		No	241 (46.80)
HIV	Positive	16 (3.11)	Blood film	Yes	302 (58.64)
	Negative	499 (96.89)		No	213 (41.36)
Anemia	Yes	45 (8.74)	Crystalline penicillin	Yes	334 (64.85)
	No	470 (91.26)		No	181 (35.15)
Heart disease	Yes	14 (2.72)	Ampicillin and gentamycin	Yes	116 (22.52)
	No	501 (78.45)		No	399 (77.48)
Chest in drawing	yes	128 (24.85)	Hydrocortisone	Yes	31 (6.02)
	no	387 (75.15)		No	484 (93.98)
Plural effusion	Yes	11 (2.14)			
	No	504 (97.86)			
Down syndrome	Yes	11 (2.14%)			
	No	504(97.86%)			

### Treatment outcomes

**Figure 1:**
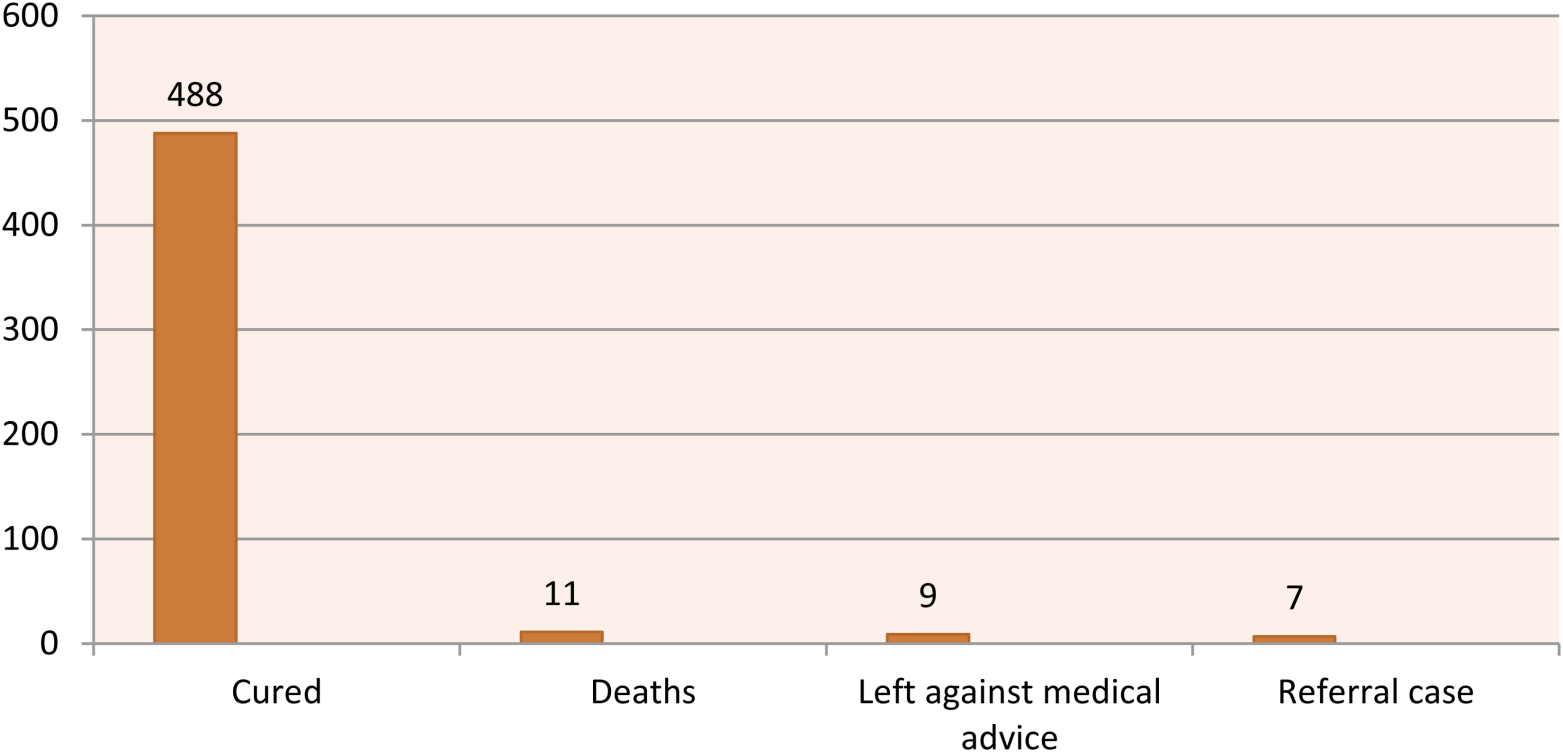
Treatment outcome of children admitted with SCAP (*n* = 526).

The majority (n = 488, 94.76%) of children recovered from SCAP, while 11 (2.14%) died, 7 (1.36%) were referred for further management, and 9 (1.75%) left against medical advice (Figure 1).

### The median hospitalization time and incidence rate of recovery from SCAP

The total follow-up time was 2,478 person-day observations and the overall recovery rate from SCAP was 19.69 per 100 person-day observations (95% CI, 18.02–21.52). A total of 149 (28.93%) patients had a prolonged hospitalization time (>5 days). The rate of prolonged hospitalization was 22.67 per 100 person-days (95% CI, 15–39).

### Overall survival function for hospitalization time

**Figure 2:**
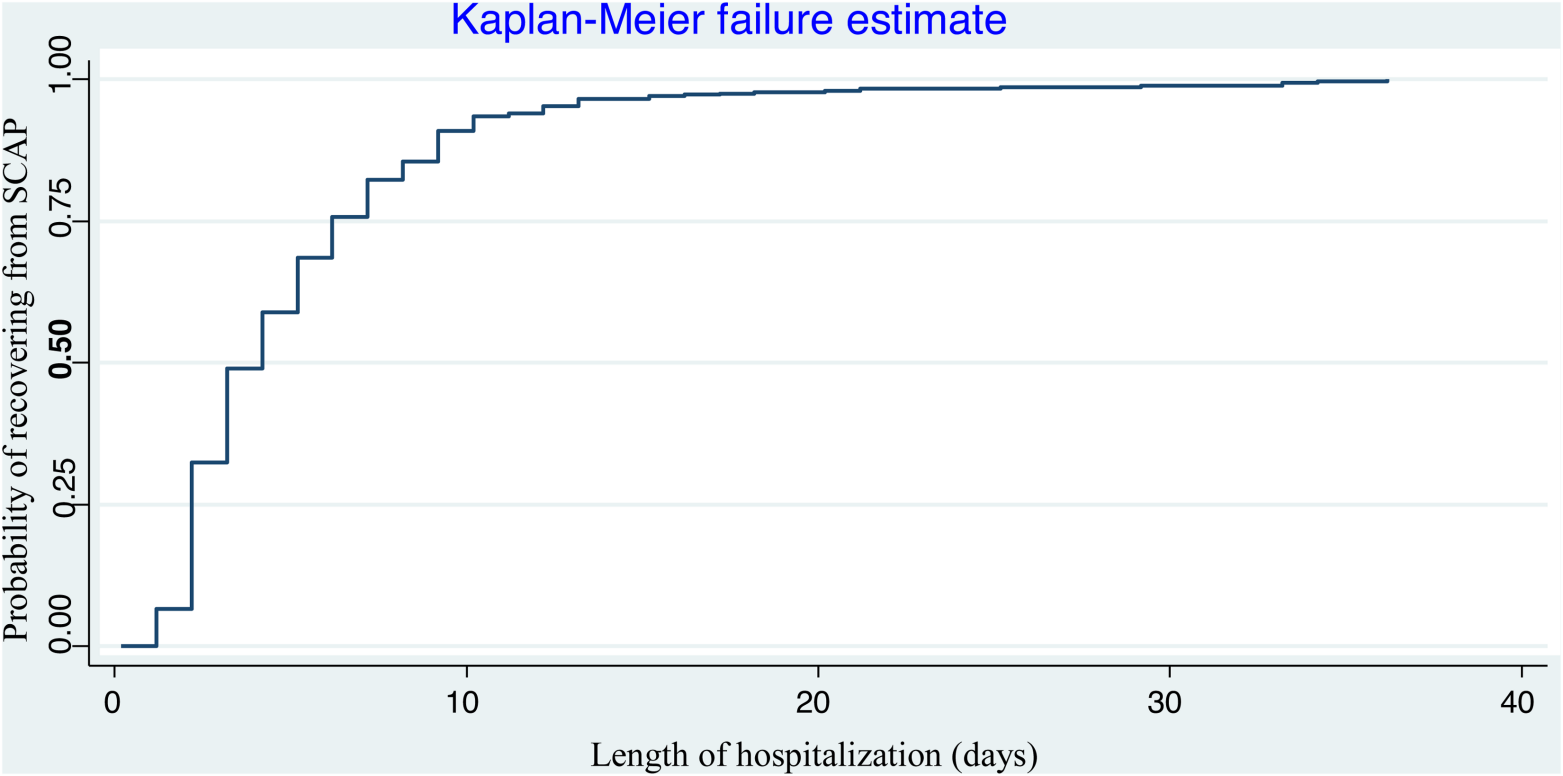
Overall survival function (95% CI-survival function) of children with SCAP admitted to Benishangul-Gumuz public hospitals.

The overall Kaplan–Meier estimate showed that the probability of recovering from severe community-acquired pneumonia increased as the follow-up days increased; the median hospitalization time was 5 days (IQR = 2–8) (Figure 2).

### Survival function and comparison of different categorical variables

Patients with a controlled facility referral source, wasting, late presentation, anemia, comorbidity, investigation of hemoglobin level and blood film, and presence of pleural effusion complications had a longer length of hospital stay or lower probability of recovering early from SCAP than the corresponding categories. All these categorical variables have a Log-rank *p* ≤ 0.05, which shows the presence of a statistically significant difference in the length of hospital stay among the corresponding categories.

### Bivariable and multivariable Cox regression analysis

In the multivariable Cox Regression model, having facility referral sources, wasting, late presentation to hospital, anemia, pulmonary effusion, no identified hemoglobin level, and no identified blood film were significantly associated with the probability of recovering from SCAP or length of hospital stay. However, health insurance, height for age, pneumothorax, HIV, heart disease, Down syndrome, meningitis, heart disease, drug regimen, complete blood count, and chest x-ray were not significant predictors of prolonged hospitalization (Table 3).

**Table 3:** Bivariable and multivariable Cox regression analysis of 2–59-month children admitted to SCAP in Benishangul-Gumuz region public hospital, Ethiopia.

Variable	Category	Recovered (%)	Censored (%)	CHR (95% CI)	AHR (95% CI)	*p*-value
Family Health insurance	Yes	242 (95.7)	11 (4.3)	0.78 (0.65–0.93)	0.93 (0.77–1.12)	0.448
	No	246 (93.9)	16 (6.1)	1	1	
Referral source	Facility referral	155 (93.9)	10 (6.1)	0.54 (0.45–0.66)	0.79 (0.63–0.98)	0.035*
	self-referral source	333 (95.1)	17 (4.9)	1	1	
WFH	Normal	435 (94.4)	26 (5.6)	1	1	0.024*
	Wasting	53 (98.1)	1 (1.9)	0.40 (0.29–.55)	0.64 (0.44–.94)	
HFA	Normal	427 (94)	27 (6)	1	1	0.224
	Stunting	61 (100)	0	0.42 (0.31–0.56)	0.77 (0.50–1.18)	
WFA	Normal	425 (94)	27 (6)	1	1	0.781
	Under weight	63 (100)	0	0.39 (0.29–0.52)	0.93 (0.58–1.51)	
Vomiting Everything	Yes	69 (94.5)	4 (5.5)	0.75 (0.58–0.97)	1.02 (0.78–1.33)	0.910
	No	419 (94.8)	23 (5.2)	1	1	
Duration of illness before admission	Early comer	348 (95.6)	16 (4.4)	1	1	<0.001*
	Late comer	140 (92.7)	11 (7.3)	0.48 (0.39–0.59)	0.67 (0.53–0.84)	
Pulmonary effusion	Yes	9 (81.8)	2 (18.2)	0.46 (0.24–0.89)	0.31 (0.15–0.66)	0.002*
	No	479 (95)	25 (5)	1	1	
Pneumothorax	Yes	9 (100)	0	0.50 (0.26–0.96)	0.72 (0.34–1.52)	0.391
	No	479 (94.6)	27 (5.4)	1	1	
HIV	Positive	13 (81.25)	3 (8.75)	0.62 (0.36–1.08)	0.84 (0.47–1.48)	0.543
	Negative	475 (95.2)	24 (4.8)	1	1	
Anemia	Yes	43 (95.6)	2 (4.4)	0.57 (0.41–0.78)	0.65 (0.46–0.90)	0.011*
	No	445 (94.7)	25 (5.3)	1	1	
Down syndrome	Yes	8 (72.7)	3 (27.3)	0.53 (0.26.07)	0.57 (0.27–1.22)	0.151
	No	480 (95.2)	24 (4.8)	1	1	
Heart disease	Yes	12 (85.7)	2 (14.3)	0.59 (0.33–1.04)	0.67 (0.36–1.24)	0.204
	No	476 (95)	25 (5)	1	1	
Meningitis	Yes	8 (100%)	0	0.56 (0.28–1.13)	0.57 (0.28–1.17)	0.127
	No	480 (94.7%)	27 (5.3%)	1	1	
Chest x-ray	Yes	171 (93.4)	12 (6.6)	1	1	0.046*
	No	317 (95.5)	15 (4.5)	0.63 (0.52–0.76)	0.81 (0.65–0.99)	
Complete blood count	Yes	220 (97.3)	6 (2.7)	1	1	0.507
	No	268 (92.7)	21 (7.3)	0.53 (0.44–0.63)	0.92 (0.72–1.12)	
Hemoglobin	Yes	268 (97.8)	6 (2.2)	1	1	<0.001*
	No	220 (91.3)	21 (8.7)	0.42 (0.35–0.50)	0.53 (0.41–0.70)	
Blood film	Yes	298 (98.7)	4 (1.3)	1	1	<0.001*
	No	190 (89.2)	23 (10.8)	0.51 (0.42–0.61)	0.65 (0.53–0.80)	
Crystalline pencil line	Yes	320 (95.8)	14 (4.2)	0.48 (0.40–2.59)	1.09 (0.82–1.50)	0.540
	No	168 (92.8)	13 (7.2)	1	1	
Ampicillin and gentamycin	Yes	107 (92.2)	9 (7.8)	0.45 (0.36–0.56)	1.40 (0.99–1.95)	0.054
	No	381 (95.5)	18 (4.5)	1	1	
Hydrocortisone	Yes	31 (100)	0	1	1	0.11
	No	457 (94.4)	27 (5.6)	0.78 (0.54–1.13)	1.49 (0.91–2.43)	

CHR, crude hazard ratio; AHR, adjusted hazard ratio.

1 = Reference.

* Statistically significant <0.05.

## Discussion

Investigating the time to recovery from SCAP and its predictors would help to reduce the extended time of hospitalization and poor treatment outcomes of pediatric patients.

The overall incidence of recovery rate (19.69 per 100 person-days) in this study was higher than that in the study conducted at Debre Markos (16.25 per 100 person-day) (19) and Gondar (13.5 per 100 person-day) (24). The discrepancy could be due to differences in a healthcare setting and in sample size as our study is in a resource-limited area and the sample size was larger than the above two studies. However, this discrepancy might be because the counter studies were conducted in referral and specialized hospitals where critical and severe cases with a lower probability of recovery were referred from other healthcare facilities.

The median time to recovery from SCAP in this study (5 days) was higher than the research findings in Gondar (3 days), Hawassa (3 days), Debre Markos (4 days), and the United States (3 days) (8, 19, 24, 25). However, the median recovery time was lower than in studies from the Netherlands (6.7 days) (9) and Tanzania (10 days) (26). These discrepancies could be due to the difference in time when the studies were conducted, the healthcare system, and the methodology of the study. Our study took place in a remote area of Ethiopia where the majority of the population lives in rural areas and in poverty. Children left the hospital against medical advice because of their parent’s inability to pay healthcare expenses, and because the healthcare system was not well organized. Our study used a survival analysis, which considers censored participants, while the study from Tanzania used a logistic regression analysis, which does not consider this.

Children who were late seeking medical treatment (presenting after 3 days of illness) from the healthcare services and those with a facility referral source on admission had a 33% and 21% risk of prolonged hospitalization time, respectively. It is congruent with the studies in Debre Markos (19) and Kenya (27). The delay in seeking medical treatment for 3 days or more from a healthcare facility increases the severity of the illness and complications, which may result in worsening progression and prolonged time to recovery. On the contrary, having a referral from another healthcare facility could show that the case was critical and that the patient might have been delayed due to issues with transportation.

The probability of recovery in malnourished children with severe wasting was reduced by 36% compared to well-nourished children. This is supported by studies conducted in Ethiopia (24) and Nepal (28). Undernourished children were more likely to have a micronutrient deficiency (29) and other related medical complications (30), leading to prolonged hospitalization time.

Children with anemia at admission or during the treatment periods were 35% less likely to recover early compared to those who did not have anemia. This agreed with the findings conducted in Hawassa (8). However, other related studies from Ethiopia at Gondar and Debre Markos reported that anemia was not a significant predictor for time to recovery (19, 24). This could be due to inter-institutional differences in strictly adhering to the earlier assessment of anemia and the difference in the prevalence of anemia in the study areas.

The presence of pulmonary effusion complications had a 69% risk of prolonging hospitalization time, which is in line with another study (22). The presence of complications, such as pleural effusion, shows the severity of the case and necessitates more advanced management, leading to more time required for treating the complications and to recovery.

Performing a laboratory test for hemoglobin at admission or during the treatment period was a significant predictor for recovery time. Children without identified hemoglobin levels were 47% less likely to recover early compared to those having an identified hemoglobin level. This was consistent with a study conducted in Morocco (31). It is possible that investigating hemoglobin levels could provide an opportunity for early intervention in children with anemia. Without a hemoglobin level, the investigation might delay the early diagnosis of anemia and thus delay patients from receiving lifesaving blood transfusion interventions, which could result in a poor prognosis (32).

Children admitted without a blood film investigation had a 35% risk of prolonged hospitalization compared to children who had a blood film investigation at admission or during the treatment period. This is in line with a study conducted in Mozambique (7). The reason might be that malaria and SCAP in admitted children aged under 5 years may show clinical similarities, making the differential diagnosis challenging, and resulting in the inability to respond to standard treatments if not identified early by blood film (33).

## Limitations of the study

The study was conducted based on children's secondary data; the primary data, such as maternal nutritional status and children's feeding habits, were not addressed.

## Conclusions and recommendations

The median hospital length of stay was longer compared to other studies. Wasting, late presentation to hospital, pulmonary effusion, anemia, the absence of identified hemoglobin level, and blood film results during treatment or at admission were factors that increased the length of hospitalization. Hence, attention should be given to the prevention of malnutrition and anemia in children, increasing early health-seeking behavior in the community. Attention should be given to complications from severe community-acquired pneumonia, such as pleural effusion, and tests for hemoglobin and blood films should be performed upon the child’s admission to hospital. Researchers recommended conducting prospective studies to include primary data from the caregivers.

## Data Availability

The original contributions presented in the study are included in the article, further inquiries can be directed to the corresponding author.
